# Decoding the exosomal secretome: stem cell-elicted microenvironmental reprogramming for anterior cruciate ligament regenerative medicine

**DOI:** 10.3389/fbioe.2025.1691651

**Published:** 2025-11-28

**Authors:** Junjie Chen, Jiayang He, Dujiang Yang, Yuhan He, Yuhao Wei, Zhiqiang Zhang, Xue Zhou, Ming Cheng

**Affiliations:** 1 Department of Rehabilitation, Jinniu District People’s Hospital of Chengdu, Chengdu, China; 2 Chengdu University of Traditional Chinese Medicine, Chengdu, China; 3 Southwest Medical University, Luzhou, China; 4 College of Integrated Traditional Chinese and Western Medicine, Southwest Medical University, Luzhou, China; 5 School of Sports Medicine and Health, Chengdu Sport University, Chengdu, China

**Keywords:** ACL rupture, inflammatory response, stem cell therapy, exosomes, neovascularization, bone homeostasis

## Abstract

The ACL rupture is a prevalent and debilitating joint injury that has garnered significant clinical and scientific interest. Surgical reconstruction is often necessary for ACL rupture, yet numerous clinical cases indicate that the outcomes of such procedures are frequently suboptimal. Research has highlighted that the treatment of ACL rupture is particularly challenging due to factors such as the inflammatory response, the formation of vascular scar tissue, and the slow healing of tendon-bone interface attachment points. These complications result in poor integration of tendon grafts within bone tunnels. Recent advancements in stem cell research have introduced new possibilities for ACL rupture treatment. However, stem cell therapy is not without its limitations, including safety concerns such as the risk of aberrant differentiation leading to oncogenesis. Exosomes, a type of extracellular vesicle secreted by stem cells, have been found to modulate immune responses, promote neovascularization, influence scar formation, and regulate bone homeostasis *in vivo*. This review seeks to systematically evaluate the therapeutic potential of stem cell-derived exosomes in the context of ACL rupture repair.

## Highlights

- ACL Rupture Challenge: Anterior cruciate ligament (ACL) ruptures are common knee injuries that often require surgical intervention, but outcomes may be suboptimal due to various complications.
- Complications of ACL Repair: Factors such as inflammatory responses, vascular scar tissue formation, and delayed tendon-bone healing hinder effective integration of grafts in surgical repairs.
- Stem Cell Research Advances: Recent advancements in stem cell therapies present new potential for improving ACL repair; however, concerns about safety and aberrant cell differentiation persist.
- Exosomes in Tissue Repair: Stem cell-derived exosomes can modulate immune responses, promote neovascularization, and regulate scar formation, offering promising therapeutic avenues for ACL repair.
- Systematic Evaluation: This review critically assesses the therapeutic potential of exosomes in enhancing ACL rupture repair, highlighting their role in tissue regeneration and functional recovery.

## Introduction

The ACL is a crucial anatomical structure that connects the femur to the tibia, serving to stabilize and control the anterior-posterior movement of the knee joint. With the increasing popularity of sports and physical fitness activities, the risk of ACL injury has steadily risen. ACL rupture typically results from traumatic hyperextension, direct impact, or unnatural movements that exert excessive stress on the ligament, leading to its complete rupture (Tian et al., 2023). Annually, approximately 200,000 patients suffer ACL injuries, making it one of the most prevalent ligament injuries in musculoskeletal disorders. Clinically, ACL reconstruction via surgical intervention remains the predominant treatment approach (Zou et al., 2023; Silvers-Granelli et al., 2017). This procedure often employs knee arthroscopy to insert a graft that replaces the damaged ligament. However, despite surgical advancements, the re-injury rate remains concerning, with an estimated 11.7% of ACL reconstructions failing and up to 94% of tendon graft reconstructions leading to re-tearing (Chen H. et al., 2021; Peng et al., 2022; Wang et al., 2021).

Successful graft healing, particularly through the regeneration of Sharpey fibers and proper integration into the bone marrow tract, is essential for effective ACL repair. The tendon-bone interface, where the ligament attaches to the bone, plays a critical role in this healing process (Lipner et al., 2014). At this interface, ligament fibers transition gradually into bone tissue, intertwining with the connective tissue on the bone’s surface. This complex attachment site comprises four distinct histological zones: organized tendon, uncalcified fibrocartilage, calcified fibrocartilage, and bone tissue (Lipner et al., 2014). However, the transition from the soft ligament to the hard bone presents challenges, as impaired neovascularization, excessive collagen fibrosis, and inflammatory responses hinder the formation of fibrocartilage at the tendon-bone junction. Consequently, these factors contribute to a heightened risk of ACL re-tearing following surgical reconstruction, driven by the fragility of the healed ligament. Given these limitations, there is a pressing clinical need for improved adjuvant therapies to mitigate the risk of ACL re-injury and enhance long-term outcomes for patients.

With the rapid advancement of bioengineering, mesenchymal stem cell (MSC) adjuvant therapy has emerged as a promising strategy for treating ACL injuries. MSCs, sourced from a wide range of tissues, offer several advantages including low cost, multipotent differentiation capacity, and immunomodulatory potential, positioning them as highly prospective candidates for ACL repair (Chen et al., 2023). Despite these benefits, MSC therapy also encounters significant limitations and challenges. Studies have reported that MSCs may exhibit immunogenicity, tumorigenic potential, and ectopic differentiation, raising critical safety concerns for their clinical application (Gleeson et al., 2015). Consequently, decellularization therapy has gained traction as a research focus within bioengineering.

Beyond the limitations of cell-based therapies, the biological environment of the injured ACL itself presents a series of interconnected hurdles that impede successful regeneration. These include: 1) a potent early inflammatory response that creates a catabolic milieu, disrupting the healing process (Zhang et al., 2022a); 2) the challenge of orchestrating controlled neovascularization to support repair without promoting fibrosis (Komro et al., 2020); 3) the inherent propensity for the formation of biomechanically inferior fibrotic scar tissue at the tendon-bone interface, rather than a regenerated, graded fibrocartilage (Zou et al., 2023); and 4) the need to provide specific osteochondral cues within the bone tunnel to guide the complex process of graft integration. Overcoming this multifaceted biological problem requires a therapeutic strategy capable of multi-targeted regulation (Śmigielski et al., 2016).

Stem cell-derived exosomes—nanoscale extracellular vesicles (30–150 nm) loaded with bioactive molecules—are formed through the inward budding of the plasma membrane, leading to early endosomes that mature into multivesicular bodies (MVBs) via ESCRT-dependent (Kowal et al., 2014) or ESCRT-independent pathways involving tetraspanins and ceramide (Babst, 2011). During maturation, intraluminal vesicles encapsulate functional biomolecules from the parent cell. MVBs are transported to the plasma membrane by Rab GTPases (e.g., Rab27a/b) (Henne et al., 2011) and release exosomes via SNARE-mediated fusion (e.g., VAMP7) (Cui et al., 2022).

Exosomes offer several advantages over direct stem cell transplantation. Firstly, they circumvent many of the technical and safety issues associated with cellular therapies, such as challenges related to cell viability, ectopic differentiation, and immune rejection (Trounson and McDonald, 2015). Secondly, exosomes can be employed as drug carriers, facilitating targeted delivery through various administration routes, including direct injection, intravenous infusion, aerosols, and topical application, thus enabling customization based on the specific therapeutic goal. Lastly, exosomes exert multifaceted therapeutic effects by releasing vesicles loaded with biologically active factors. These factors promote cellular proliferation, attenuate inflammation, stimulate angiogenesis, and ultimately contribute to tissue repair and regeneration.

**FIGURE 1:**
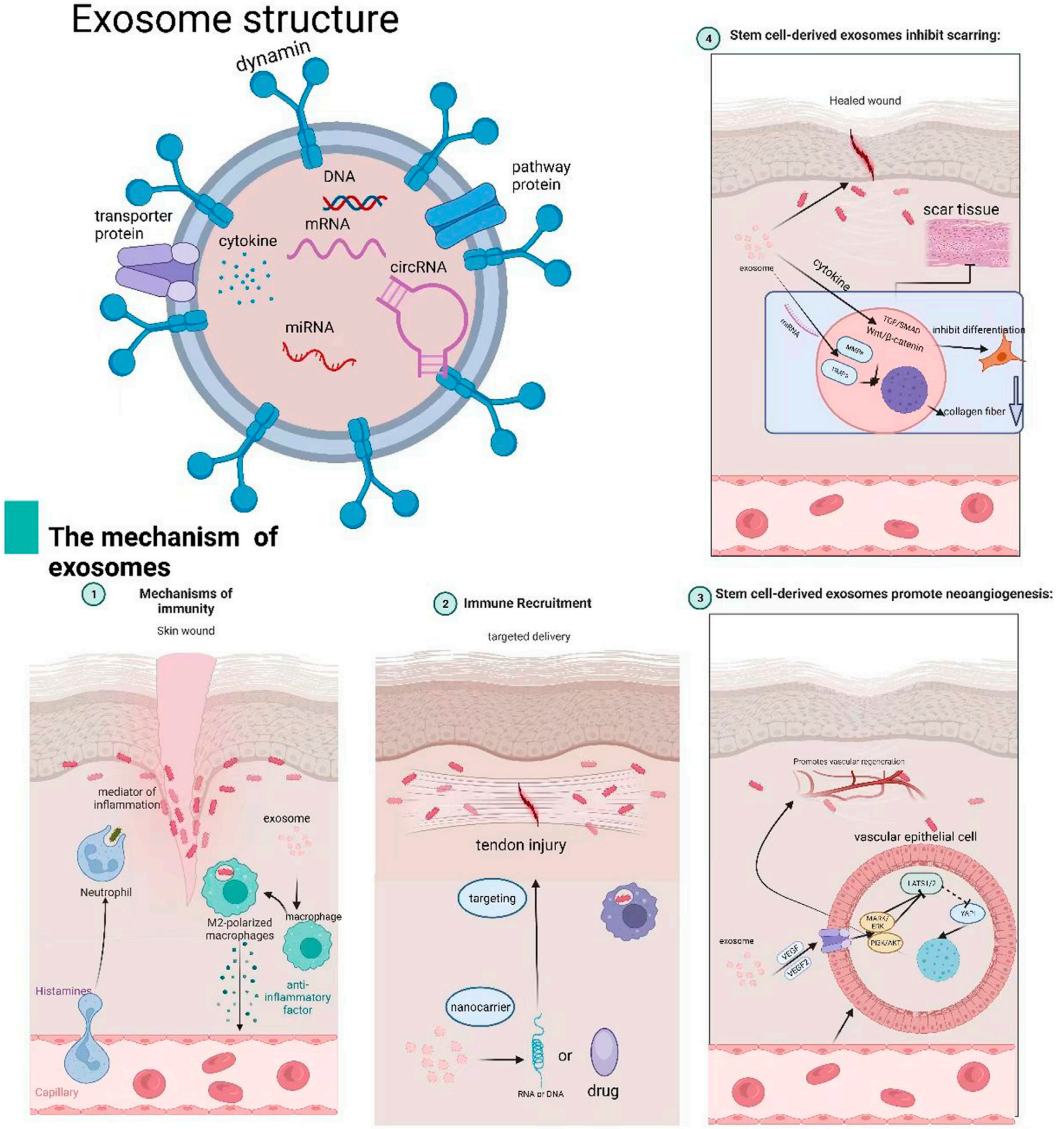
Exosome structure and mechanisms in tissue repair.

Given these attributes, exosome-based therapies derived from stem cells hold considerable promise in the treatment of ACL ruptures by directly addressing these biological challenges. As natural carriers of bioactive molecules, exosomes can be engineered to deliver a precise combination of anti-inflammatory miRNAs (to quench early synovitis), pro-angiogenic and anti-angiogenic factors (to fine-tune vasculature), anti-fibrotic signals (to inhibit scar formation), and osteochondral morphogens (to direct tissue differentiation). This review aims to explore the current clinical challenges and limitations of ACL injury treatments, evaluate the potential of stem cell and exosome-based therapies in tissue engineering, and elucidate the underlying mechanisms through which exosomes contribute to ACL repair and regeneration (Figure 1).

## Anatomy of the ACL

The ACL is a pivotal structure within the knee joint, serving primarily to stabilize the knee and prevent anterior translation of the tibia relative to the femur. Anatomically, the ACL originates from the posterior aspect of the medial surface of the lateral femoral condyle and courses obliquely downward and anteriorly to insert on the anteromedial aspect of the tibial plateau. It is composed of two primary bundles: the anteromedial (AM) bundle and the posterolateral (PL) bundle. These bundles twist in a helical configuration as they extend posteriorly, laterally, and superiorly, terminating in a fan-shaped attachment on the posterior-superior aspect of the medial surface of the lateral femoral condyle. The femoral attachment site of the ACL covers an area approximately 20 mm by 10 mm at its maximal diameter, and the ligament itself measures approximately 4 cm in length (ranging from 3.7 to 4.1 cm). Each bundle contributes differently to knee stability depending on the degree of knee flexion or extension.

## Pathologic changes in ACL injuries

Pathologically, an ACL injury typically manifests as a partial or complete tear of the ligament, resulting from either acute trauma or repetitive mechanical stress. The injury leads to a disruption in the ligament’s structural integrity, characterized by the tearing or rupture of the collagen fiber meshwork that constitutes the ligament. This disruption severs the connections between collagen fibers, accompanied by local damage to fibroblasts and extracellular matrix (ECM) components (Hasegawa et al., 2012). Table 1 response to the injury, fibroblasts undergo apoptosis and necrosis, while neutrophils, macrophages, and other inflammatory cells rapidly accumulate at the site, releasing a cascade of inflammatory mediators that exacerbate tissue damage. Simultaneously, matrix metalloproteinases (MMPs) within the ECM become activated, further degrading collagen and other matrix components, thereby impeding the natural tissue repair process.

**TABLE 1:** Summary of in vivo studies using stem cell-derived exosomes in various rat models.

Model	Exosome source	Administration regimen	Assays and evaluations	Key findings	Safety assessment	References
Rat TMJ-OA model	BMSC-exos	20 μg/joint (protein); days 1, 3, 5 post-modeling	Elisa: NO, MMP13; WB: s-GAG, Col2; HE: synovitis; macrophage M2 polarization (CD206^+^) *in vitro*	MSC-exos promote joint repair via adenosine/AKT/ERK/AMPK signaling	No joint toxicity/foreign body reaction; cell viability >90%; normal short-term physiology	27
Rat knee OA model	miR-140-5p-overexpressing hUSC-Exos	100 μg/dose (protein); Weekly × 4 weeks	Behavior: Joint mobility, thermal pain threshold; ELISA: IL-1β, TNF-α; WB: Col2, aggrecan; Safranin-O	hUSC-140-exos deliver miR-140-5p to downregulate VEGFA, inhibit vascular invasion, protect cartilage	No excessive inflammation/rejection; normal liver/kidney function	47
Rat diabetic skin wound model	BMSC-exos	FHE hydrogel +50 μL exosomes (1 × 10^9^ particles/mL); Applied immediately and every 3 days for 2 weeks	Wound closure rate, re-epithelialization; HE: granulation tissue and collagen; IHC: CD31, α-SMA	FHE hydrogel exhibits self-healing, injectability, antibacterial and pH-responsive exosome release	No infection, allergy, or tissue necrosis	48
Rat tendon defect model	TDSC-exos	50 μg (protein); single implantation with sustained-release scaffold	CCK-8 and scratch assay: Proliferation/migration; HE and masson: Healing/collagen; RT-qpcr/WB: miR-144-3p/ARID1A	TDSC-exos deliver miR-144-3p to enhance tendon cell proliferation, migration, and early healing	p-HA scaffold biocompatible; cell viability >95%; no adhesion/limitation	49
Rat chronic rotator cuff tear	KGN-pretreated BMSC-exos	100 μg/mL, 50 μL/rat; days 1, 7, 14 post-op; SAH sustained release up to 96 h	Safranin-O/fast green, Col2 IHC: Cartilage; sirius red: collagen maturity; RT-qPCR: SOX9, aggrecan, Col2a1	KGN priming enhances exosome chondrogenic activity, promoting cartilage regeneration and collagen maturation	No hemorrhage, calcification, or abnormal proliferation	50
Rat ACL reconstruction model	Hypoxia-pretreated BMSC-exos	Dose not specified; single injection + hydrogel sustained release ≥14 days	Micro-CT: BV/TV; HE and safranin-O/fast green: Healing interface; IHC: CD31/EMCN; biomechanics: Load/stiffness	Hypo-exos promote H-type vessel formation via HIF-1α/miR-126, accelerating tendon-bone healing	No hydrogel-related edema, synovitis, or tumor-like lesions; normal immunity	51
Rat ACL reconstruction model	BMSC-exos	200 μg (total protein); single implantation during surgery	Micro-CT: Tunnel widening; HE and masson: Collagen integration; IF: BMP7/Smad5 expression	BMSC-exos + cartilage fragments activate BMP7/Smad5 signaling, suppress tunnel widening, enhance integration	No rejection, foreign body response, or fibrosis; normal joint function	73

In addition to cellular and matrix disruptions, vascular permeability in the injured region increases, leading to plasma leakage, cellular extravasation, and the formation of hematomas and edema. In the initial stages of injury, this results in a pronounced inflammatory response characterized by swelling, pain, and hematoma formation.

## Immune mechanisms of stem cell-derived exosomes *in vivo*: effects of the inflammatory response on ACL injury

The immune response to ACL injury is complex, with inflammation occupying a central role. Inflammation constitutes the body’s non-specific defence mechanism against injury, aimed at eliminating damaging factors and initiating tissue repair. Following injury, pro-inflammatory cytokines such as TNF-α, IL-1, and IL-6 are released, activating signalling pathways including NF-κB and MAPK. This promotes the expression of inflammatory genes and induces macrophage polarisation towards the M1 phenotype. M1 macrophages play a pivotal role in early repair processes, including the clearance of tissue debris and the promoetion of collagen deposition (Wynn and Vannella, 2016).

Berberine (BBR) has demonstrated anti-inflammatory effects in a rat model of adjuvant-induced arthritis (animal study, preclinical evidence). In this study, BBR was administered orally at doses of 40, 80, and 160 mg/kg for 14 days. It significantly reduced paw swelling, inflammatory cell infiltration, and joint destruction, while modulating macrophage polarization from M1 to M2 phenotype by downregulating M1 markers (iNOS, TNF-α, IL-1β, IL-6) and upregulating M2 markers (Arg1, IL-10, TGF-β1). These effects were mediated through the AMPK/NF-κB pathway, as BBR enhanced AMPK activity and suppressed phosphorylation of p65 and IκBα, as well as COX-2 expression (Zhou et al., 2019). Further *in vitro* studies confirm BBR suppresses galectin-3 expression via the AMPK/NF-κB pathway in macrophages, thereby attenuating activation (Pei et al., 2019). Moreover, in a randomized, double-blind clinical trial of RA patients, the JAK inhibitor tofacitinib reduced synovial levels of MMP-1, MMP-3, and multiple chemokines, an effect associated with inhibition of STAT1/STAT3 phosphorylation (Boyle et al., 2015).

However, as tendon injuries progress into the healing phase, an excessive inflammatory response can be detrimental, leading to tissue fibrosis, scarring, and delayed healing. Thus, a balanced anti-inflammatory response is crucial during this phase to ensure optimal tissue repair (Kim et al., 2017; Weinfeld, 2014). Thus, timely regulation of inflammation during the healing phase is critical. In a rat tendon injury model, aspirin mitigated inflammation and fibrosis by inhibiting the JNK/STAT-3 signalling pathway, thereby reducing the risk of re-rupture (Wang Y. et al., 2019). Further studies indicate that modulating pathways such as PTEN/PI3K/AKT and MAPK can alleviate inflammation, improve biomechanical properties, and promote tendon healing (Wang Y. et al., 2019).

## Anti-inflammatory mechanisms of stem cell-derived exosomes

Exosomes play a significant role in regulating the body’s inflammatory response by encapsulating and delivering anti-inflammatory molecules and signaling proteins. These EVs modulate the amplitude and duration of inflammation through various mechanisms. Zhang et al. provided preclinical evidence from a rat TMJ-OA model and *in vitro* chondrocyte cultures, demonstrating that MSC exosomes promoted joint repair by activating the adenosine/AKT/ERK/AMPK signaling axis. This mechanism counteracted IL-1β-induced damage by enhancing s-GAG synthesis and inhibiting the production of nitric oxide and MMP13 (Zhang S. et al., 2019). Inhibition of these pathways via AKT or ERK phosphorylation inhibitors reduced exosome-mediated cell proliferation and migration. This underscores the crucial role of exosome CD73 in mediating these effects, as demonstrated by CD73 inhibitor AMPCP and adenosine receptor antagonist theophylline (Zhang S. et al., 2018).

In addition to their effects on joint repair, MSC exosomes modulate macrophage polarization, shifting macrophages from the pro-inflammatory M1 phenotype to the anti-inflammatory M2 phenotype. Zhao et al. provided preclinical evidence from a murine model of myocardial ischemia/reperfusion and *in vitro* studies, demonstrating that MSC-derived exosomes (MSC-Exo) polarized macrophages via the delivery of miR-182, which inhibited TLR4 expression to promote M2 polarization and reduce inflammation (Zhao et al., 2019).

Exosomes also exert direct effects on T cells and B cells, contributing to their immunomodulatory potential. T-cells and B-cells play a synergistic role in the anti-inflammatory process through interaction and information exchange: T-cells direct B-cells to differentiate and produce specific antibodies by secreting cytokines, while B-cells activate T-cells through antigen presentation, which together can achieve effective clearance of pathogens and precise regulation of inflammatory responses, ensuring that the immune response is both efficient and does not cause excessive damage to the host tissues. *In vitro* mechanistic studies have shown that exosomes carrying active CD73 protein can suppress T cell activity via the adenosineergic pathway, thereby reducing inflammation (Kerkela et al., 2016). Preclinical studies in murine aGVHD models and *in vitro* T cell cultures demonstrated that BM-MSC-derived EVs inhibit CD3^+^ T cell activation, an effect attributed to their unique miRNA profile (e.g., miR-125a-3p) (Fujii et al., 2018). Similarly, studies in a rat heart transplant model and co-culture systems showed that exosomes from IDO-overexpressing BMSCs (IDO-BMSCs) enhanced immunomodulation by increasing Tregs, reducing CD8^+^ T cells and pro-inflammatory cytokines, and elevating anti-inflammatory cytokines like IL-10 (He et al., 2018; He et al., 2020). Additionally, MSC-exosomes delivering miRNA-181a were shown to regulate T cells, thereby reducing myocardial ischemia-reperfusion (I/R) injury through anti-inflammatory effects (Wei et al., 2019).

Although much is known about the immunosuppressive effects of exosomes on T cells, less is understood regarding their impact on B cells. Evidence from *in vitro* co-culture studies using B cells from healthy donors and plasma-derived exosomes indicates that exosomes can increase the expression of checkpoint receptors PD-1 and LAG3 on B cells, thereby inhibiting B cell function. This effect was observed with exosomes from both healthy individuals and patients, suggesting a natural suppressive role for circulating exosomes (Schroeder et al., 2020).

While stem cell-derived exosomes offer great potential in immunoregulation, they also present certain challenges. Research in this field is complicated by the lack of standardized experimental conditions and preparation methods, making it difficult to compare results across studies. Future research needs to focus on systematically studying exosomes from various sources and identifying the specific mechanisms through which they regulate inflammation. Although significant progress has been made in understanding the signaling pathways involved, a more comprehensive and detailed understanding of these pathways is required. Additionally, further studies in in vivo models of inflammation are needed to verify the therapeutic effects of exosomes and advance their clinical applications.

## Role of targeted transport in the treatment of acl rupture

Drug delivery methods, such as targeted carriers and controlled release technologies, remain in the research phase, and their clinical application faces significant challenges. The feasibility and efficacy of these approaches require further exploration to determine their potential in preventing ACL re-tear injury.

Stem cell-based drug delivery has emerged as a promising approach in the field of tissue engineering. Due to their inherent properties of migration and homing, stem cells can localize to injured sites and facilitate targeted drug delivery. Following injury, the SDF-1/CXCR4 pathway becomes activated, guiding stem cells to the injury site. SDF-1 expression increases in damaged tissues, creating a chemotactic gradient that attracts circulating CD34 (+) progenitor cells to the injury site (Lau et al., 2011). This mechanism is also supported by platelet-derived SDF-1, which regulates stem cell adhesion and differentiation into endothelial progenitor cells, promoting tissue repair (Stellos et al., 2008).

Stem cells not only migrate to injury sites but can also serve as drug carriers, providing sustained drug release over an extended period. This reduces the frequency of drug administration and enhances drug stability, bioavailability, and efficacy (Liu Z. et al., 2020). Preclinical studies in murine models of myocardial infarction have demonstrated the potential of engineered cell-mimicking systems. For instance, synthetic MSCs (synMSC) fabricated from PLGA microparticles and MSC membranes promoted angiogenesis and alleviated left ventricular remodeling (Luo et al., 2017). Similarly, platelet-inspired nanocells (PINCs) combine prostaglandin E2-modified platelet membranes and cardiac stromal cell factors to target ischemic heart tissue, thereby activating endogenous stem/progenitor cells and promoting angiogenesis during myocardial ischemia/reperfusion (I/R) injury (Su et al., 2019).

However, stem cell transplantation procedures carry inherent risks, including surgical trauma, infection, bleeding, and complications related to anesthesia. The long-term effects of stem cells, such as tumor formation and tissue malformation, also remain uncertain, along with potential interactions between stem cells and drugs (Gleeson et al., 2015; Moll et al., 2022; Zhang et al., 2017).

Exosomes offer an alternative therapeutic strategy, with their stable biological nanoparticle structure and ability to carry bioactive molecules such as proteins and nucleic acids. Exosomes have shown potential as drug delivery vehicles, offering enhanced targeting and efficacy. Preclinical studies in tumor xenograft models have demonstrated the efficacy of engineered nanovesicles for targeted therapy. Zhang et al. reported that biofunctionalized liposome-like nanovesicles (BLNs) showed superior antitumor efficacy over clinical liposomal doxorubicin in a HER2-overexpressing mouse model (Zhang P. et al., 2018). Similarly, Garofalo et al. used bioluminescence imaging in nude mouse models to confirm that exosomes loaded with oncolytic virus and paclitaxel could specifically target tumors and enhance anti-tumor effects (Garofalo et al., 2019).

Exosomes have also shown promise in tendon-bone injury repair. For instance, Evidence from rotator cuff tear models—a related tendon-bone injury—shows that purified exosome products (PEP) enhanced healing, suggesting potential applicability to ACL repair (Ren et al., 2021; Han et al., 2022). In knee joint anti-inflammatory therapy, human urine-derived stem cell exosomes (hUSC-140-Exos) exhibited increased secretion of ECM components, such as collagen II and aggrecan, while inhibiting apoptosis (Liu Y. et al., 2022). However, it must be explicitly stated that these models provide only indirect evidence and conceptual analogies for ACL repair, with their clinical translation readiness yet to be validated in the ACL context.

Presently, the specific application of exosomes in ACL rupture repair remains at the exploratory frontier, with their mechanisms of action far from elucidated. Future research must transcend analogies derived from other tissues, committing to generating data from the ACL itself to unlock its true potential for targeted therapy.

## Extended-release exosome loading

In the field of modern medicine, the development of drug delivery systems has seen significant advancements, with exosome-based systems gaining attention for their potential in both targeted and sustained drug release. Exosomes, as biological nanoparticles, offer a unique mechanism for the storage and controlled release of therapeutic agents, enabling prolonged therapeutic effects that hold promise for disease treatment.

A particularly promising innovation in this area involves the combination of exosomes with materials like hydrogels to enhance their sustained release capabilities. For example, FHE hydrogels demonstrate multifunctional properties, including rapid self-healing, injectable shear-thinning behavior, efficient antibacterial activity, and bioactive exosome release. Wang C. et al. (2019) demonstrated that FHE@exo hydrogels significantly improved the healing of diabetic full-thickness skin wounds. The study showed that FHE@exo hydrogels outperformed both exosomes and FHE hydrogels used individually, indicating that the sustained release of exosomes in combination with hydrogels can synergistically promote wound healing in diabetic patients.

Similarly, sustained-release exosome systems have shown potential in treating tendon injuries. For instance, Song et al. employed a photopolymerized hyaluronic acid (p-HA) scaffold equipped with tendon-derived stem cell exosomes (TDSC-Exos) to treat tendon injuries in a rat model. This pHA-TDSC-Exos scaffold served as a controlled release system for treating tendon defects, with miR-144-3p in TDSC-Exos promoting tendon cell proliferation and migration by targeting the AT-rich interactive domain 1A (ARID1A) (Song et al., 2022).

In a rat model of chronic rotator cuff tear, a controlled laboratory study (Level: preclinical) demonstrated that exosomes derived from kartogenin-preconditioned MSCs (KGN-Exos), when delivered via a sustained-release sodium alginate hydrogel, significantly promoted tendon-to-bone healing. This was evidenced by enhanced cartilage formation, collagen maturation, and superior biomechanical properties compared to untreated exosomes (Cai et al., 2023).

In the treatment of ACL injuries, extended-release exosomes have also demonstrated potential benefits. Zhang T. et al. (2022) showed that exosomes derived from hypoxically cultured bone marrow mesenchymal stem cells (Hypo-Exos) enhanced graft osseointegration after ACL reconstruction. The study revealed that Hypo-Exos, when adhered to hydrogels, provided continuous release around the graft site for at least 14 days. Bone volume/total volume ratio (BV/TV) measurements of the femur and tibial bone tunnel areas, as well as grafted bone, indicated significantly better outcomes in the Hypo-Exos group compared to the control and normoxic exosome (Norm-Exos) groups (P < 0.05).

These findings highlight the critical role of exosome loading in enhancing tissue repair, particularly in the context of ACL injuries. However, further research is necessary to fully elucidate the mechanisms underlying exosome-mediated healing in these applications.

## Stem cell-derived exosomes promote neoangiogenesis

The application of exosomes in vascular regeneration holds great promise, particularly in promoting wound healing and addressing tendon injuries such as rotator cuff and ACL injuries. Exosomes derived from pluripotent stem cells, specifically mesenchymal stem cell exosomes (hiPSC-MSC-Exos), have been shown to stimulate the formation of new blood vessels and accelerate their maturation at wound sites (Zhang J. et al., 2015).

Further research has identified angiopoietin-2 (Ang-2) within human umbilical cord mesenchymal stem cell-derived exosomes (hucMSC-Exos). Treatment with hucMSC-Exos enhances Ang-2 expression in wound areas and in human umbilical vein endothelial cells (HUVECs), contributing to tube formation and angiogenesis (Liu et al., 2021). Additionally, hucMSC-Exos promote the nuclear translocation of β-catenin and increase the expression of proliferative nuclear antigen, cyclin D3, N-cadherin, and β-catenin while decreasing E-cadherin expression. This highlights the critical role of Wnt/β-catenin signaling in hucMSC-Exos-induced angiogenesis (Zhang B. et al., 2015).

A series of preclinical studies in diabetic rat models and *in vitro* endothelial cell cultures have consistently demonstrated the pro-angiogenic effects of MSC-derived exosomes. Teng et al. reported that hucMSC-exosomes promoted wound healing by increasing CD31 and VEGF expression and reducing TNF-α (Teng et al., 2022). Similarly, exosomes encapsulated in PVA/alginate nanohydrogels (exo@H) were shown to upregulate VEGF via the ERK1/2 pathway, accelerating diabetic wound repair (Zhang Y. et al., 2021).

Furthermore, evidence from both animal models and mechanistic cell studies indicates that pharmacological preconditioning enhances exosome efficacy. Exosomes from pioglitazone-pretreated MSCs (PGZ-Exos) promoted angiogenesis by activating the PI3K/AKT/eNOS pathway^ (Zhang T. et al., 2022)^. Likewise, exosomes from atorvastatin-pretreated MSCs (ATV-Exos) exerted pro-angiogenic effects via the AKT/eNOS pathway, mediated by upregulation of miR-221-3p (Hu et al., 2021; Yu et al., 2020).

Exosomes also contribute to bone tunnel healing after ACL reconstruction. Hypo-Exos have been found to increase the abundance of H-type blood vessels within the bone tunnel area at week two post-surgery, significantly enhancing graft integration (Zhang T. et al., 2022). Preclinical evidence from rat femoral fracture models and *in vitro* studies demonstrates that uMSC-Exos promote angiogenesis and bone healing via VEGF upregulation (Zhang Y. et al., 2019). The underlying mechanism involves Hypo-Exos promoting angiogenesis via the miR-126 and SPRED1/RAS/ERK signaling pathways. (mechanistic *in vitro* studies)Knockdown of hypoxia-inducible factor 1 (HIF-1α) diminishes these effects, suggesting that HIF-1α is crucial for Hypo-Exos-mediated cardiovascular production and fracture healing (Liu W. et al., 2020). Furthermore, exosome HMGB1 from myelo-depleted MSCs under hypoxic conditions has been reported to increase angiogenesis through the JNK/HIF-1α pathway (Gao et al., 2021).

However, in tendon-bone healing, such as after ACL injury, excessive vascularization may adversely affect tissue repair. Uncontrolled vascular growth, driven by an od re-tear rates in ACL injuries. More research is required to fully understand the mechanisms behind angiogenesis in ACL healing and to develop strategies that balance vascular growth to optimize long-term healing outcomes.

## Stem cell-derived exosomes inhibit scarring

Injury to the ACL often results in scar tissue formation, which can hinder recovery. The ACL is crucial for maintaining knee stability and function. When damaged, the body initiates a self-healing response, often involving scar tissue formation. However, this scar tissue is typically less elastic and more rigid than normal tissue, which can increase the risk of re-injury (Zou et al., 2023; Ateschrang et al., 2018). Scar tissue, being a substitute structure, lacks the functional qualities of the original tissue, leading to increased stiffness and reduced joint performance.

Recent studies have shown that exosomes, which are small EVs, play a significant role in regulating scarring. Exosomes can inhibit scar formation through various mechanisms, including anti-inflammatory effects, regulation of fibrosis, modulation of angiogenesis, and degradation of ECM components (Zhou et al., 2023; Bian et al., 2022; Tutuianu et al., 2021). For instance, Wang C. et al. (2019) (Xu et al., 2023) demonstrated that FHE@exosome (FHE@exo) hydrogel injections promoted skin regeneration with fewer scars, suggesting that exosomes effectively inhibit scar tissue formation. Dinh et al. (Dinh et al., 2020) found in animal models that exosomes derived from lung stem cells (LSC-exo) reduced collagen accumulation and myofibroblast proliferation, mitigating lung fibrosis and scar tissue formation in models of bleomycin and silica-induced fibrosis. Exosomes also inhibit scarring by targeting specific cellular pathways. For example, adipose-derived stem cell exosomes (ADSC-Exos) effectively inhibit the proliferation and migration of fibroblasts, reducing the expression of collagen type I and III (Col1, Col3), α-SMA, and other fibrotic markers while increasing SIP1 levels, thereby improving hypertrophic scar fibrosis (Li et al., 2021). In another study, ADSC-conditioned medium (ADSC-CM) reduced collagen deposition and scarring through the p38/MAPK signaling pathway *in vitro*, *ex vivo*, and *in vivo* models (Li et al., 2016). Additionally, studies combining patient samples and mouse models show that microRNA-33 released by bone marrow mesenchymal stem cell-derived exosomes (BMSC-Exos) inhibits the IL-2/ST214 axis, alleviating skin fibrosis (Xie et al., 2023).

Exosomes have also shown promising results in preventing scarring after tendon injuries. Exosomes derived from mesenchymal stromal cells (MSCs) have been reported to mimic the M2 macrophage phenotype, promoting tendon remodeling and reducing scar formation in Achilles tendon injuries (Chamberlain et al., 2021). Tendons treated with tendon stem cell-derived exosomes (TSC-Exos) exhibit more organized and continuous tissue structure, suggesting that TSC-Exos help regulate the ECM and inhibit scarring (Zhang M. et al., 2020). Preventing scar tissue formation at the tendon-bone junction is critical for successful ACL repair. Early research suggests that BMSC-Exos may enhance tendon-to-bone healing by upregulating cartilage gene expression and boosting the BMP7/Smad5 signaling axis. In a rat model of ACL reconstruction, BMSC-Exos combined with cartilage fragments significantly reduced femoral tunnel width, suggesting improved healing with less scar formation at the tendon-bone interface (Zhang et al., 2023). The balance between angiogenesis and scar formation is another key factor in tissue repair. While new blood vessels provide oxygen and nutrients to repair tissues, excessive angiogenesis can lead to tissue fibrosis and scarring (Guillamat-Prats, 2021; An et al., 2021). Managing this balance is a significant challenge in ACL repair. Animal study evidence shows that FEP dressings combined with exosomes (FEP@exo) have shown promise in diabetic wound models by promoting cell proliferation, granulation tissue formation, and re-epithelialization, while reducing scar tissue formation and promoting the regeneration of skin appendages (Wang M. et al., 2019).

Exosomes not only promote angiogenesis but also possess antifibrotic properties, mediated by the release of matrix metalloproteinases (MMPs). This dual functionality makes exosomes a potent therapeutic option for reducing scarring while accelerating wound healing (Bian et al., 2022). Overall, exosomes hold immense potential in preventing scar formation and improving healing outcomes in tendon and ligament injuries.

In the field of ACL injury repair, exosome therapy has demonstrated significant advantages in tendon-bone healing and anti-inflammatory scar formation by virtue of its unique molecular regulatory mechanisms. Exosome therapy has a more precise and comprehensive regulatory capability than traditional methods such as PRP, MSC transplantation, graft augmentation surgery and scaffold-mediated tissue engineering.

PRP therapy relies on growth factors (e.g., PDGF, VEGF, TGF-β1) released upon platelet activation to drive healing by promoting fibroblast and bone progenitor cell proliferation, but high concentrations of TGF-β1 may trigger haphazard deposition of collagen fibres at a later stage, leading to disturbed collagen alignment, which in the long term may exacerbate fibrosis and scar hardness. The anti-inflammatory mechanism of MSC transplantation can only weakly regulate the activity of immune cells through limited cytokines (e.g., IL-10), and its ability to regulate macrophage polarisation (M1/M2 transformation) is insufficient to inhibit excessive scar proliferation caused by persistent inflammation (Le et al., 2018); MSC transplantation can directly replenish the functional cells of the tendon bone interface through the multidirectional differentiation potential of the stem cells. However, transplanted cells have a low homing efficiency and short survival time, and their direction of differentiation is significantly affected by the local microenvironment (e.g., hypoxia, mechanical stress), which often results in an imbalance between over-differentiation of osteoblasts and under-differentiation of tendon fibres. The amount of soluble factors (e.g., PGE2, IDO) secreted by immunomodulation-dependent cells is easily interfered by the inflammatory environment, and may be ineffective in the acute inflammatory phase due to the overdose of pro-inflammatory factors (e.g., TNF-α), resulting in fluctuating anti-inflammatory effects and unstable inhibition of scar formation (Wang et al., 2020; Liu J. et al., 2022); graft augmentation surgery and scaffold-mediated tissue engineering are more reliant on physical support and structural guidance: the former provides mechanical stability through the implantation of autologous/allogeneic grafts, and the other provides mechanical stability through the implantation of autologous/allografts. Graft-enhanced surgery and scaffold-mediated tissue engineering rely more on physical support and structural guidance: in the former, the implantation of autologous/allogeneic grafts provides mechanical stability, but the nature of healing is the gradual replacement of grafts by host tissues, and the accompanying foreign-body reaction (especially in the case of allogeneic grafts) will continue to activate inflammatory pathways (e.g., NF-κB), which will increase the risk of excessive fibrotic scarring (Uma et al., 2009); in the latter, the use of biological scaffolds to mimic extracellular matrices can be loaded with growth factors, but it is difficult to fully match the rate of scaffold degradation and cellular adhesion with the pace of human tissue regeneration. Although the latter uses biological scaffolds to mimic the extracellular matrix, the degradation rate and cell adhesion of the scaffold material are difficult to match the regeneration rhythm of human tissues, and the traces of local microenvironmental “artificial intervention” may be too strong, leading to the abnormal activation of fibroblasts, and triggering the disorder of scar tissue.

In contrast, exosomes, as nanoscale vesicles secreted by stem cells, carry miRNAs, proteins, and other active components, and promote tissue repair through multi-targeted synergistic effects. In tendon-bone healing, the miRNAs (e.g., miR-140, SOX-9) and growth factors (BMP-2, VEGF) precisely induced the differentiation of tendon stem cells to fibroblasts, and at the same time, promoted osteogenesis of bone progenitor cells, constructed the “tendon-fibrocartilage-bone” gradient interface, and enhanced biomechanical integration; In anti-inflammatory scar formation, exosomes block M1-type macrophage activation by inhibiting the NF-κB pathway, induce M2-type anti-inflammatory phenotype, reduce the release of pro-inflammatory factors, such as TNF-α and IL-6, and regulate TGF-β1 signalling to inhibit excessive deposition of type I collagen and promote the formation of flexible scars by type III collagen, so as to achieve the orderly collagen metabolism, inhibit fibrosis and scar formation and optimize the healing process (Li et al., 2022). The effect is further optimised by inhibiting fibrosis and scar formation.

In conclusion, exosome therapy shows more precise and comprehensive therapeutic effects in tendon-bone healing and anti-inflammatory scars, and has the advantages of non-invasiveness and stability, so it has a greater potential for clinical application in the treatment of ACL, and it is expected to bring a better prognosis and fewer postoperative complications for patients.

## Conclusion and prospect

In summary, mesenchymal stem cell-derived exosomes (MSCs-exos) show promising potential in the treatment of ACL injuries by enhancing the repair process. These exosomes release biological factors that interact with various mechanisms, including immune modulation, inhibition of scarring, and promotion of angiogenesis, to support ACL recovery. The unique targeting capabilities and sustained release properties of MSCs-exos contribute positively to the healing of ACL injuries (Arabpour et al., 2021). Anti-inflammatory molecules such as interleukin 10 (IL-10), transforming growth factor β (TGF-β), and chemokine CCL1 within exosomes help to reduce inflammation at the injury site (An et al., 2021). Additionally, exosome-derived cytokines promote the expression of factors like TGF-β3 and MMP3, which inhibit the excessive synthesis and deposition of collagen fibers, ultimately reducing scar formation by reorganizing collagen in scar tissue (Wang et al., 2017). Furthermore, exosomes release vascular endothelial growth factor (VEGF), matrix metalloproteinases (MMPs), and microRNAs that stimulate the proliferation and migration of endothelial cells, thereby accelerating vascularization and promoting ACL repair (Olejarz et al., 2020).

However, the timing and extent of exosome-mediated repair depend on the severity of the injury and the local microenvironment, making it a challenge to balance these multiple repair mechanisms effectively. Finding the optimal regulatory balance remains a significant hurdle in promoting ACL repair. Following ACL injury, the resolution of inflammation is critical for effective tissue repair. The initial inflammatory response is initiated by the infiltration of immune cells, including macrophages, neutrophils, and lymphocytes. These cells release inflammatory mediators such as IL-6, TNF-α, and IL-1β, which facilitate the clearance of necrotic tissue and pathogens, thereby creating a favorable environment for healing. Although chronic inflammation can lead to tissue degeneration, a tightly regulated inflammatory resolution process promotes the degradation and remodeling of the ECM by modulating the activity of fibroblasts and tenocytes, thereby establishing the foundation for successful tissue repair (Brigant et al., 2018; Yang and Xia, 2021). Consequently, maintaining a controlled inflammatory response through exos is essential for the repair of ACL injuries. Despite these advantages, exosome-based therapies face substantial challenges. The easily degradable nature of exosomal proteins and nucleic acids necessitates stringent preparation, storage conditions, and special processing methods (Zhang Y. et al., 2020). The isolation of MSC-derived exosomes (MSC-exos) is typically achieved through several techniques, including ultracentrifugation, density gradient centrifugation, immunoaffinity capture, nanofiltration, and ultrafiltration. While various methods for isolating MSC-exos exist, none has been universally accepted as the gold standard. Ultracentrifugation is one of the most widely used techniques, which utilizes a stepwise gradient centrifugation process to separate MSC-exos from cell culture supernatants or biological samples (Boing et al., 2014). The procedure begins with low-speed centrifugation to remove cells and larger debris, followed by medium-speed centrifugation to eliminate smaller fragments and microparticles. Subsequently, high-speed centrifugation is employed to isolate exosomes, which are precipitated at the bottom of the centrifuge tube. The exosome pellet is then collected and resuspended to obtain purified MSC-exos. Ultracentrifugation is a well-established and efficient exosome isolation technique that effectively separates exosomes from other cellular debris, providing high purity with relatively simple operation and low cost, making it suitable for a variety of sample sources (Chen J. et al., 2021; Xu et al., 2023). However, this method also has some drawbacks, including a lengthy process, potential structural alterations due to high centrifugal forces, the need for specialized and costly equipment, possible residual impurities that may cause structural damage or aggregation, and co-isolation with lipoproteins. These factors may limit its application in certain experimental contexts. Furthermore, prolonged or improper storage can significantly diminish the biological activity of exosomes (Coughlan et al., 2020). Their storage conditions are notably stringent, primarily due to their susceptibility to structural damage and functional alterations in adverse environments. The integrity of the exosomal membrane and the bioactive components it contains—such as proteins and nucleic acids—are particularly sensitive to variables including temperature, pH, and storage duration. Moreover, using phosphate-buffered saline (PBS) as a diluent for exosomes can significantly reduce their viability over short periods. However, studies have shown that human albumin and trehalose-containing PBS (PBS-HAT) can improve exosome preservation at −80 °C, though further research is needed to confirm its efficacy as an optimal storage condition (Boing et al., 2014).

Exosomes also hold great potential for targeted therapy and systemic drug delivery, but significant challenges remain regarding drug-loading techniques and modes of administration. Exosomes can be administered via intravenous injection or subcutaneous injection, and the administration route plays a critical role in treatment efficacy. Intravenous injection enables the rapid systemic distribution of exosomes but may trigger immune responses or lead to their swift clearance by the circulatory system (Kamerkar et al., 2017). Nasal administration, a non-invasive approach, is particularly suitable for treating central nervous system disorders, although its absorption efficiency may present certain limitations. Local injection allows for achieving high concentrations in target tissues, typically offering a favorable safety profile; however, its applicability is confined to localized lesions (Zhang X. et al., 2021). Oral administration, being more convenient and patient-friendly, faces challenges such as degradation in the gastrointestinal tract, necessitating strategies to enhance its bioavailability (Ju et al., 2013). Therefore, selecting an appropriate delivery route requires a comprehensive evaluation of the disease type, target tissue requirements, patient compliance, and the physicochemical properties of exosomes, aiming to strike an optimal balance between safety and feasibility. Intra-articular injection is the most common method for treating knee joint conditions, and has shown promising results in rat models of knee arthropathy, although further experiments are needed to determine the optimal delivery method for various conditions.

As drug carriers, exosomes can be loaded either pre-secretively or post-secretively, making them a highly promising drug delivery system due to their unique structural and biological properties. In pre-secretory drug loading, the loading of drugs by endogenous cellular mechanisms, such as transgenesis or co-culture, enables efficient natural loading and targeted modification with uniform drug distribution and high biocompatibility. However, this method requires complex cell culture and transfection techniques, and has restricted drug selection and loading, limited control over loading efficiency, and the potential to disrupt membrane proteins that are critical to exosome function, and is particularly ineffective for certain drugs that may affect cell survival (Barile and Vassalli, 2017).

Post-secretory drug loading, where a therapeutic agent is added to exosomes, can lead to exosome aggregation, membrane damage and low yield. In contrast, postsecretory drug loading, which involves direct loading of drugs by physical or chemical means, offers the advantages of flexibility, high loading capacity, and rapid manipulation, but may damage exosome membranes, affecting their stability and function, as well as poorly distributing the drug uniformly and potentially introducing additional toxicity. Overall, presecretory loading is suitable for molecules such as RNA or proteins that require high drug stability, while postsecretory loading drugs are more suitable for rapid loading of small molecule compounds or for improving loading efficiency in specific applications (Ha et al., 2016). Post-isolation drug loading strategies for BMSC-derived exosomes encompass physicochemical methods (co-incubation, electroporation), genetic engineering, and surface functionalization (Ha et al., 2016; Kimiz-Gebologlu and Oncel, 2022; Sadeghi et al., 2023), with application-specific optimization for shoulder injury therapeutics requiring combinatorial anti-inflammatory and angiogenic effects. Method selection necessitates rigorous optimization based on drug physicochemical properties (molecular weight, solubility) and therapeutic parameters (target specificity, pharmacokinetics). Macromolecular angiogenic factors preferentially employ sonication/electroporation for structural preservation and enhanced permeability (Zeng et al., 2023), while gene-based therapeutics utilize electroporation for nucleic acid integrity maintenance. Chemical conjugation enables precision targeting, with polymer-based co-delivery systems proving effective for multi-agent combinations (e.g., anti-inflammatory/angiogenic cocktails) requiring sustained release profiles (Xu et al., 2020). Small-molecule anti-inflammatories predominantly utilize passive loading via incubation/freeze-thaw cycles due to molecular stability (Xi et al., 2021), though inherent exosomal membrane hydrophilicity poses challenges for lipophilic drug encapsulation. To overcome this limitation, advanced nanotechnological approaches have emerged: 1) Hybrid vesicle systems employing liposome-exosome membrane fusion for hydrophobic cargo transfer; 2) Ultrasound-mediated transient membrane permeabilization enhancing lipid-soluble drug entrapment through acoustic modulation of membrane fluidity (Shi et al., 2023). These innovations address critical biophysical barriers in exosomal drug loading while preserving vesicle integrity and bioactivity. Improvements in exosome drug-loading technology are critical for advancing exosome-based targeted drug delivery systems, and future research is essential to address these challenges.

The clinical translation of MSCs-Exos confronts several critical challenges, chiefly characterized by a paucity of human clinical trials, predominant reliance on animal model-derived data, and absence of standardized clinical protocols. The paucity of human clinical investigations constitutes the principal impediment to therapeutic development, with current research (as of October 2023) remaining predominantly confined to preclinical exploration through *in vitro* analyses and animal experimentation. Although preclinical evidence suggests therapeutic potential in tissue regeneration and immunomodulation–exemplified by neurorestorative effects observed in murine stroke models (Xin et al., 2013) – these findings lack robust clinical validation in human populations. The translational barrier primarily stems from fundamental interspecies disparities in injury response mechanisms, particularly evident in central nervous system pathologies where rodent models inadequately recapitulate human pathophysiology. Exosomes carry MHC-I-like molecules of matricellular origin on their surface, which may trigger a host immune response but are significantly less immunogenic than intact cells (Li et al., 2025). For example, exosomes from allogeneic MSCs did not trigger significant T cell activation in rodent models (Zhang et al., 2022c). Notably, exosomes carry pathogen-associated molecular patterns (PAMPs) or damage-associated molecular patterns (DAMPs) that may activate intrinsic immunity and need to be excluded from contamination by strict quality control (Shen et al., 2022). This persistent dependence on preclinical data introduces substantial uncertainty regarding clinical efficacy, underscoring the imperative for rigorous human trials to bridge the translational gap between experimental models and therapeutic applications.
